# Kinetics and Mechanism of Cyanobacteria Cell Removal Using Biowaste-Derived Activated Carbons with Assessment of Potential Human Health Impacts

**DOI:** 10.3390/toxins16070310

**Published:** 2024-07-09

**Authors:** Irina Kandić, Milan Kragović, Sanja Živković, Jelena Knežević, Stefana Vuletić, Stefana Cvetković, Marija Stojmenović

**Affiliations:** 1 “Vinča” Institute of Nuclear Sciences, National Institute of the Republic of Serbia, University of Belgrade, 11351 Belgrade, Serbia; irina.kandic@vin.bg.ac.rs (I.K.); m.kragovic@vin.bg.ac.rs (M.K.); sanjaz@vin.bg.ac.rs (S.Ž.); 2 Institute of Public Health of Serbia “Dr. Milan Jovanović Batut”, dr Subotića Starijeg 5, 11000 Belgrade, Serbia; jelena_jovanovic@batut.org.rs; 3 Department of Microbiology, Faculty of Biology, University of Belgrade, Studentski trg 16, 11000 Belgrade, Serbia; stefana.d@bio.bg.ac.rs (S.V.); stefana.cvetkovic@bio.bg.ac.rs (S.C.)

**Keywords:** activated carbon, biowaste, cyanobacteria, adsorption mechanism, cytotoxicity, genotoxicity, health impact

## Abstract

Harmful cyanobacteria blooms and the escalating impact of cyanotoxins necessitates the effective removal of cyanobacteria from water ecosystems before they release cyanotoxins. In this study, cyanobacteria removal from water samples taken from the eutrophic Aleksandrovac Lake (southern Serbia) was investigated. For that purpose, novel activated carbons derived from waste biomass—date palm leaf stalk (P_AC), black alder cone-like flowers (A_AC), and commercial activated carbon from coconut shell (C_AC) as a reference were used. To define the best adsorption conditions and explain the adsorption mechanism, the influence of contact time, reaction volume, and adsorbent mass, as well as FTIR analysis of the adsorbents before and after cyanobacteria removal, were studied. The removal efficiency of P_AC and A_AC achieved for the applied concentration of 10 mg/mL after 15 min was ~99%, while for C_AC after 24 h was only ~92% for the same concentration. To check the safety of the applied materials for human health and the environment, the concentrations of potentially toxic elements (PTEs), the health impact (HI) after water purification, and the toxicity (MTT and Comet assay) of the materials were evaluated. Although the P_AC and A_AC achieved much better removal properties in comparison with the C_AC, considering the demonstrated genotoxicity and cytotoxicity of the P_AC and the higher HI value for the C_AC, only the A_AC was further investigated. Results of the kinetics, FTIR analysis, and examination of the A_AC mass influence on removal efficiency indicated dominance of the physisorption mechanism. Initially, the findings highlighted the superior performance of A_AC, with great potential to be globally commercialized as an effective cyanobacteria cell adsorbent.

## 1. Introduction

Cyanobacteria are a highly diverse group of prokaryotes, thus exhibiting a wide range of morphologies, from unicellular to filamentous forms. They have the ability to form dominant populations in ecosystems, thus leading to the occurrence of cyanobacterial blooms. These blooms, which commonly occur in freshwater environments, disrupt ecosystems and pose a global public health risk. The persistence of mass populations of cyanobacteria is believed to be influenced by the rapid growth of the human population [1]. In recent decades, cyanobacteria have gained significant attention due to the production of secondary metabolites known as cyanotoxins, which have detrimental effects on human health. Cyanobacteria in freshwater may produce a range of toxins, such as microcystins, saxitoxins, nodularins, cylindrospermopsin, and anatoxin-a [2]. Cyanotoxins can be categorized into groups such as hepatotoxins (microcystins and nodularin), cytotoxins (cylindrospermopsin, including neurotoxic, hepatotoxic, and genotoxic), neurotoxins (anatoxin-a, homoanatoxin-a, anatoxin-a(S), saxitoxin, and beta-methylamino-L-alanine), and dermatotoxins (Lyngbya toxins and aplysiatoxins) [2,3,4]. Cyanobacteria inhabit various ecosystems and possess highly effective ecophysiological adaptations and strategies that allow them to colonize a wide range of habitats, including extreme environments [5]. Anthropogenic activities, including agricultural drainage and industrial and municipal waste, have the potential to affect water quality [6]. Consequently, these activities can create ecosystems that are significantly impacted by excessive nutrient input, thus leading to eutrophication, water level variations, desiccation, and salinization [7]. Small lakes and ponds are particularly susceptible to cyanobacterial blooms, as they are sensitive ecosystems with longer water retention times, increased nutrient accumulation, and higher trophic status that promote blooming. The research on small lakes has raised awareness regarding the importance of freshwater biodiversity, its role in ecosystem services, and its vulnerability to anthropogenic disturbances [8]. The most common species of cyanobacteria in freshwater environments include *Microcystis* spp. (Kützing), *Rhapidiopsis raciborskii* (formerly *Cylindrospermopsis raciborskii*) (Woloszynska and Aguilera et al.), *Planktothrix rubescens* (De Candolle ex Gomont) (Anagnostidis and Komárek), *Synechococcus* spp. (Nägeli), *Planktothrix agardhii* (De Candolle ex Gomont) (Anagnostidis and Komárek), *Gloeotrichia* spp. (P.G. Richter), *Anabaena* spp. (Bory de Saint-Vincent ex Bornet and Flahault), *Lyngbya* spp. (Agardh ex Gomont), *Aphanizomenon* spp. (A. Morren ex Bornet and Flahault), *Nostoc* spp. (Vaucher, 1888, ex Bornet and Flahaul), certain *Oscillatoria* spp. (Vaucher ex Gomont), *Schizothrix* spp. (Kützing ex Gomont), and *Synechocystis* spp. (Sauvageau) [9].

Phytoplankton, with their short lifespans and rapid reactions to variations in the aquatic ecosystem, serve as early alert indicators of water pollution [10]. Therefore, monitoring eutrophic lakes is crucial, as it provides valuable information regarding changes in water quality and the potential presence of toxin-producing organisms, such as cyanobacteria. Continuous monitoring is essential to ensure the safety of water for irrigation purposes or recreational activities, and it should be treated with utmost seriousness [11]. To safeguard public health, the World Health Organization (WHO) has established critical control points to mitigate the risks associated with cyanotoxins. The WHO (2003) has provided guidelines for both drinking water and recreational water bodies to ensure public health safety by setting permissible levels of cyanobacteria density [12]. For drinking water, it is recommended that the presence of cyanobacteria cells should not exceed 2000 cells/mL to deem it suitable for consumption. Regarding recreational purposes, the water is considered safe when the cyanobacteria cell count remains below 20,000 cells/mL (Level 1). However, if the count reaches 100,000 cells/mL, it becomes mandatory to conduct cyanotoxin analyses (Level 2). In cases where the cell count surpasses 100,000 cells/mL, indicating the presence of a cyanobacteria bloom, all activities on or in the waterbody are prohibited (Level 3). These recommended thresholds serve as indicators to evaluate the suitability of water for various purposes and to help protect individuals from potential hazards associated with cyanotoxins.

The occurrence of cyanobacteria taxa in water bodies in Serbia has shown an increasing trend over the past two decades [13]. The Aleksandrovac Lake, located in the southern part of Serbia, is an artificial reservoir where cyanobacteria blooms have been observed [14]. The map of the sampling site is presented in the paper by Kandić et al., 2022 [15]. The lake was previously utilized for irrigation, sports, recreational activities, and fishing. However, due to the intensifying eutrophication process, the lake has become conducive to cyanobacteria blooms. The presence of cyanobacteria blooms has necessitated the suspension of these activities to protect public health. Therefore, the lake has been selected for monitoring and the removal of cyanobacteria cells.

Instances of significant fish mortality in Aleksandrovac Lake were documented in 2008 and 2012, thus coinciding with the occurrence of cyanobacteria blooms [14,16]. Despite the complete refilling of the lake with fresh water in 2010, rapid eutrophication processes once again led to cyanobacteria blooms. Several potential factors contribute to fish mortality, including changes in lake water pH, decreased oxygen concentration, and the presence of cyanobacteria and their toxins [14]. The blooming of the invasive species *Rhapidiopsis raciborskii* was detected in 2012, and it was associated with fish mortality [11]. However, during that period, microcystin, cylindrospermopsin, and saxitoxin were not detected. According to Svirčev et al. [16], other metabolites produced by cyanobacteria may have been responsible for the fish mortality. Another potential factor contributing to fish mortality could be the presence of potentially toxic elements (PTEs) in the waterbody. However, Milošković et al. [8] conducted an examination of the fish tissue for PTEs and excluded their involvement in the fish mortality in Aleksandrovac Lake. The study indicated that there was no significant risk to human health from consuming fish, as the levels of PTEs in the fish muscle (meat) were lower compared to those in the liver and gills. Nevertheless, consuming fish from Aleksandrovac Lake is not recommended due to the presence of cyanobacteria, which are well-known environmental contaminants capable of harming the entire ecosystem. One of the major concerns regarding PTEs is that once they are released into the aquatic ecosystem, they can disperse and accumulate in various components of the aquatic biota, including the flora and fauna [8]. Some of these elements are highly toxic to human health, even at low concentrations. Therefore, the presence of cyanobacteria and the potential for PTE accumulation highlight the importance of taking precautions and implementing measures to protect the ecosystem and human health.

One of the main challenges in removing cyanobacteria cells is the potential for cell lysis, which can result in the release of cyanotoxins into the water. Merely removing the cells from the water is not sufficient if the cells become damaged, as this can lead to toxin release. Park et al. [17] reported a method for cyanobacteria removal without cell lysis using an adsorption technique with a polyethyleneimine (PEI)-modified chitosan–waste biomass composite fiber (PEI-CBF). Conventional water treatment processes include coagulation, flocculation, sedimentation, filtration, and sludge removal [18]. Adsorption is a suitable technique, as it does not require additional energy or materials and can effectively recover or remove various pollutants [18,19]. The applicability of adsorption continues to expand each year, which is driven by the development of more efficient and cost-effective adsorbents [20,21]. Activated carbon, due to its high porosity, is also well-suited for adsorption and the removal of contaminants from polluted water [17,22]. There have been recently research projects for microcystin removal using activated carbon, like pinewood-based, which exhibited the high adsorption capacity and the most favorable conditions for the biological removal of MC-LR [23]. However, it is crucial to ensure that the use of activated carbons does not have any negative impacts on the environment or human health. Attention should be given to the possibility of dissolving potentially toxic elements that may be present in these materials. Materials containing heavy metals can release these metals into the environment during use, thus posing potential health risks, and assessing these risks is crucial for determining material suitability. Several studies have reported noncarcinogenic and carcinogenic risks from heavy metals and pesticides via ingestion, inhalation, and dermal contact [24]. Long-term exposure to heavy metals can adversely affect the immune, nervous, and endocrine systems, thus leading to cancer or impairments in both adults and children [25]. Moreover, these toxic elements can enter the food chain and eventually accumulate in fish tissue through the process of bioaccumulation. Additionally, it is important to test the cytotoxic and genotoxic effects of these materials. Conducting cytotoxicity and genotoxicity studies is an essential initial step in determining the potential toxicity of the applied materials. Cytotoxicity refers to the ability of a substance to induce cell death. Hence, if the materials exhibit high toxicity levels in vitro, they are likely to have partial toxicity in an in vivo environment. Emphasizing water quality is crucial, as it is closely linked to the health of local residents [25].

The preliminary research presented in the paper by Kandić et al. [15] showed that the adsorption of cyanobacteria using activated carbon materials derived from biowaste, specifically date palm leaf stalk (*Phoenix dactylifera*; P_AC) and black alder cone-like flowers (*Alnus glutinosa*; A_AC), can be successfully achieved at a concentration of 250 mg/25 mL of contaminated water after 24 h. In order to compare all the investigated parameters, a commercially available activated carbon material, made from coconut shell—AquaSorb^®^ HSL (8 × 30 MESH) (C_AC)—was also applied and investigated. The structural and chemical properties of the obtained activated carbons have been characterized and are also described in the paper by Kandić et al. [15].

In order to obtain systematic and more precise results regarding the adsorption of cyanobacteria by using mentioned activated carbon materials, as well as the removal mechanism, in this paper, research was carried out in two series. The first research series included the following:

(1) The influence of contact time on the removal of cyanobacteria cells at a concentration of 250 mg/25 mL of contaminated water taken in August 2021 by using P_AC, A_AC, and C_AC activated carbon materials. The experimental results were further analyzed using different kinetic models to explain the kinetics of cyanobacteria cell removal;
(2) The evaluation of potentially toxic elements (PTEs) in the activated carbons P_AC, A_AC, and C_AC, as well as their health impact (HI), including estimation of the chronic daily intake (CDI) and hazard quotient (HQ);
(3) The evaluation of cytotoxicity and DNA damage caused by testing all three applied activated carbons (P_AC, A_AC, and C_AC) using the MTT assay and the Comet assay, respectively.

After completing the initial series of experiments, it was concluded that activated carbon P_AC demonstrated toxicity (results presented in Section 2.5), while C_AC exhibited limited efficacy in removing cyanobacteria (results presented in Section 2.2). In accordance with these results, the second series of research that continued on contaminated water was carried out in the next year, including only the application of activated carbon A_AC, when the cyanobacterial bloom started again and increased the number of cyanobacteria, specifically in June 2022:

(1) Examination of the influence of mass of the adsorbent (A_AC) and the volume of contaminated water on cyanobacteria cell removal from water collected in June 2022;
(2) Establishment of the mechanism of the cyanobacteria cell removal using activated carbon A_AC.

Therefore, the main goal of the paper was to define the kinetics and mechanism of cyanobacteria removal prior to cell wall damage and the release of cyanotoxins, thus identifying the most suitable removal method that is both environmentally safe and that ensures public health. Given the increasing prevalence and consequences of cyanobacteria blooms, in this study, we evaluated the effectiveness of activated carbon materials as an adsorbent for purifying water from eutrophic lakes by removing cyanobacteria cells.

## 2. Results and Discussion

### 2.1. Qualitative and Quantitative Cyanobacteria Analyses of Aleksandrovac Lake

The changes in phytoplankton diversity in a lake can be the response of changes in environment, such as hydrological transformations, climate changes, newly present invasive species, etc. The qualitative and quantitative phytoplankton analyses of Aleksandrovac Lake showed the dominance of cyanobacteria in the lake during the examined period: August 2021 and June 2022 (Table 1).

The total number of cyanobacteria cells in August of 2021 was 5,093,067 cells/mL [15]. In June 2022, the total number of cyanobacteria cells was 3,725,651 cells/mL. Such extremely high numbers of cyanobacteria cells indicate that cyanobacteria blooms are present in this water. According to the WHO regulations regarding the potential health risks, this water body is at Level 3 [12]. The most dominant species in August 2021 and June 2022 was *Raphidiopsis raciborskii*, which numbered at 1,330,286 cell/mL and 2,752,423 cell/mL. 

Out of the 15 taxa of cyanobacteria identified in August 2021 and June 2022, 6 of them are known to be producers of cyanotoxins. Specifically, *Microcystyis aeruginosa* and *Pseudanabaena limnetica* are responsible for producing anatoxins, *Rhapidiopsis raciborskii* produces cylindrospermopsin, and *Microcystyis aeruginosa*, *Microcystis flos-aquae*, *Oscillatoria limosa*, and *Rhapidiopsis raciborskii* are associated with the production of microcystins and saxitoxins [15]. This does not necessarily mean that they are incapable of producing any cyanotoxins or other secondary metabolites commonly found in cyanobacteria. In the situation where various cyanobacterial species prevail within water bodies, this makes it challenging to identify a clear dominant taxon, and the identification of cyanotoxin producers can be very difficult [26]. Research on this topic continues to be conducted around the world. For instance, recent findings by Daniels et al. [27] revealed the novel and unexplored nature of *Limnothrix* (strain AC0243) poisoning due to its recently identified toxicity. Despite its potential, this strain is commonly disregarded as a source of poisonings, which is often associated with *R. raciborskii*. Nonetheless, trials have demonstrated the strain’s acute toxicity to mammals [27].

**Figure 1:**
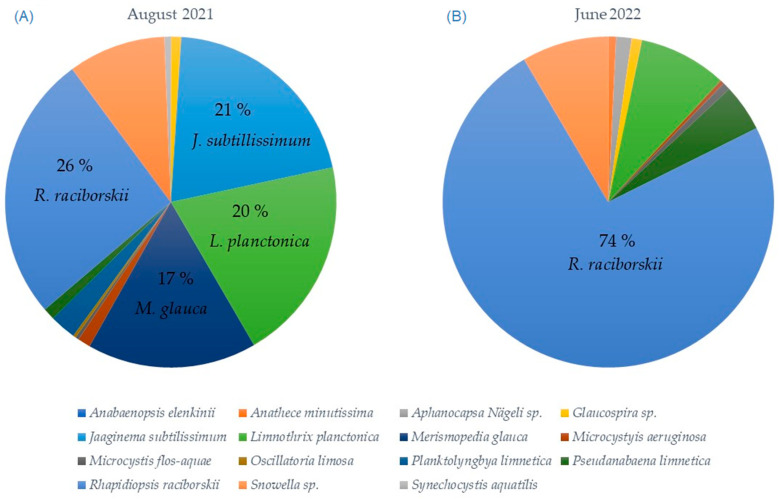
The quantitative representation of cyanobacteria in Aleksandrovac Lake in (**A**) August 2021 and (**B**) June 2022.

The *R. raciborskii* species accounted for 26% of the total number of cyanobacteria, as depicted in Figure 1A. The subsequent species in terms of abundance were *Jaaginema subtillissimum* at 21%, *Limnothrix planctonica* at 20%, and *Merismopedia glauca* at 17% (Figure 1A). However, in June 2022, the dominance of *R. raciborskii* significantly increased, thus representing a staggering 74% of all the cyanobacteria observed in Aleksandrovac Lake (Figure 1B). This indicates a substantial shift in the composition of the cyanobacteria community, with *R. raciborskii* emerging as the predominant species during that period.

These comparisons highlight the dynamic nature of cyanobacterial populations in Aleksandrovac Lake. The fluctuations in cell counts indicate the influence of various environmental factors on the growth and decline of different species. The growth of cyanobacteria is heavily influenced by physical factors, particularly the prevailing local weather conditions [28,29]. Monitoring and understanding these population dynamics are crucial for assessing the lake’s ecological health and managing the potential risks associated with cyanobacterial blooms. Detected cyanobacteria blooms with the species that are potential cyanotoxin producers indicates the necessity of more detailed biomass monitoring of potentially toxic cyanobacteria, as well as the analysis of cyanotoxins in the water [30].

### 2.2. Influence of Contact Time on the Cyanobacteria Cells Removal

**Figure 2:**
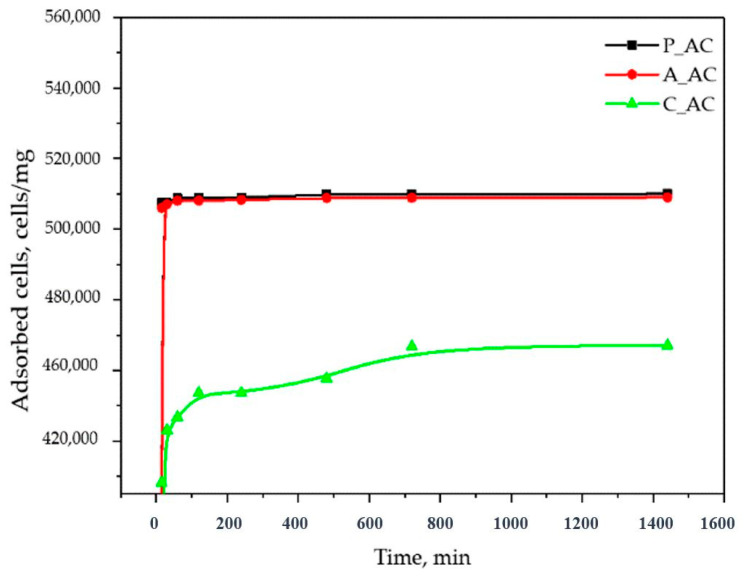
Kinetics of cyanobacteria removal by P_AC, A_AC, and C_AC.

The removal of cyanobacteria cells using activated carbons P_AC, A_AC, and C_AC was studied over a time interval from 15 min to 24 h. The experiments were conducted by using 250 mg of the adsorbents in 25 mL of contaminated water. The obtained results are present in Figure 2 and Table 2, Table 3 and Table 4. The results for 24 h were preliminarily published in a paper by Kandić et al. [15].

For the activated carbon obtained from date palm leaf stalk (P_AC), the results show that only ~18,000 cells/mL were present in the water after 15 min. According to the WHO, that means that type of water is in Level 1 and is suitable for recreational purposes [12]. The water could be suitable for drinking after 12 h when the number of cyanobacteria cells was under 2000 cells/mL. The results after 24 h show that the water is safe for usage, with a relatively small number of cyanobacteria cells (~700 cells/mL; Table 2).

After the treatment with P_AC, the effectiveness of the treatment could be observed, as several species were completely removed within just 15 min. These species include *Anabaenopsis elenkinii*, *Anathece minutissima*, *Merismopedia glauca*, *Microcystyis aeruginosa*, *Microcystis flos-aquae*, *Oscillatoria limosa*, *Planktolyngbya limnetica*, and *Synechocystis aquatilis*. This is particularly significant, because *M. aeruginosa*, *M. flos-aquae*, and *O. limosa* are known to potentially produce microcystins, which are toxins of concern.

Among the species that remained after the treatment, there were two colonial forms: *Aphanocapsa* sp. and *Snowella* sp. *Snowella* sp. were removed after 2 h of treatment. *Aphanocapsa* sp. was removed after 4 h of treatment, thus indicating its susceptibility to P_AC. The removal of *Aphanocapsa* sp. is significant due to its potential production of microcystins.

Among the species that also remained after the treatment, there were trochal forms such as *Glaucospira* sp., *Jaaginema subtilissimum*, *Limnothrix planctonica*, *Pseudanabaena limnetica*, and *Rhapidiopsis raciborskii*. *Ps. limnetica* was removed after 8 h of treatment. Of particular concern was the high number of *R. raciborskii* before the treatment. Due to its abundance and its potential production of cylindrospermopsin, it should be carefully monitored. However, the treatment with P_AC significantly reduced the number of *R. raciborskii* cells from 1,330,286 cells/mL to 104 cells/mL, thus achieving nearly 100% removal (Table 2).

From the presented results, it can be concluded that the P_AC demonstrated high effectiveness in removing various species of harmful cyanobacteria. Several species, including those capable of producing microcystins, were completely eradicated. Overall, the results demonstrate the strong efficacy of P_AC in mitigating harmful cyanobacterial blooms and reducing associated risks to aquatic ecosystems.

The activated carbon A_AC showed that after 30 min only about 16,000 cells/mL were presented in the water. That means that the water could be suitable for usage in recreational purposes according to WHO [12]. In the water after the 24 h treatment with A_AC, it contained about 600 cells/mL of cyanobacteria cells, which mean that the water is safe for drinking according to WHO [12] (Table 3).

Upon treatment with the A_AC, several species were completely removed within just 15 min, including *Anabaenopsis elenkinii*, *Anathece minutissima*, *Aphanocapsa* sp., *Microcystyis aeruginosa*, *Microcystis flos-aquae*, *Planktolyngbya limnetica*, and *Synechocystis aquatilis*. These species were effectively eliminated by the A_AC treatment.

Among the colonial forms, *Merismopedia glauca* was completely removed after 30 min of the A_AC treatment. Another colonial species, *Snowella* sp., required a longer treatment duration and was completely removed after 12 h. The ability to effectively remove these colonial species demonstrates the efficacy of A_AC against such forms.

However, among the remaining species after treatment, there were trochal forms, including *Glaucospira* sp., *Jaaginema subtilissimum*, *Limnothrix planctonica*, *Oscillatoria limosa*, *Pseudanabaena limnetica*, and *Rhapidiopsis raciborskii*. Although the trend for these species demonstrated a gradual decrease over time, only *Oscillatoria limosa* was completely removed within 1 h of treatment. Notably, *R. raciborskii* showed a higher removal efficiency with the A_AC compared to the P_AC. The number of cells decreased significantly after just 15 min, but it varied throughout the experiment, thus reaching 98 cells/mL after 24 h (Table 3).

Overall, the A_AC treatments demonstrated effectiveness in removing various species; they displayed varying degrees of success against different forms and microcystin producers. The complete removal of certain species and the substantial reduction in cell numbers highlight the potential of these treatments in managing harmful cyanobacterial blooms.

The commercial activated carbon material showed a significantly lower cyanobacteria removal rate in comparison with the P_AC and A_AC. After the 15 min treatment, the number of cyanobacteria cells per mL was more than 810,000, while after 24 h of treatment, the number of cyanobacteria cells was about 422,000 cells/mL (Table 4). That means that although the commercial material adsorbed a large amount of cyanobacteria cells, the number of cells in the water was still extremely high, and the water would neither be safe for use, nor for recreational purposes, nor for drinking, according to the WHO [12].

The application of the material C_AC resulted in the complete removal of several species after 24 h of treatment, including *Anabaenopsis elenkinii*, *Anathece minutissima*, *Microcystis aeruginosa*, *Microcystis flos-aquae*, *Oscillatoria limosa*, *Planktolyngbya limnetica*, *Snowella* sp., and *Synechocystis aquatilis*. These species were effectively eliminated, thus demonstrating the potential of C_AC in combating harmful cyanobacterial blooms.

However, there were remaining species that are known potential producers of cyanotoxins, namely *Aphanocapsa* sp. (247 cells/mL), *Pseudanabaena limnetica* (10,584 cells/mL), and *Rhapidiopsis raciborskii* (5916 cells/mL). These species pose a concern due to their ability to produce toxins that can impact water quality and ecosystem health. The presence of these species highlights the need for further treatment or management strategies to address their potential harmful effects (Table 4).

Among the remaining species, *Merismopedia glauca*, a colonial species, exhibited the highest density after treatment, with a removal efficiency of only 56% after 24 h (Table 4). It is important to note that the colonial form of this species varied from sample to sample, and the number of cells within colonies was not consistent across all samples. This variability in the colonial structure may have contributed to the lower removal efficiency observed for *M. glauca.*

Initially, the AC surface might get saturated with cells, thus leading to apparent desorption. Over time, the rearrangement or breakdown of biofilm-like structures could expose more surface area for new cells to adsorb. The typically unstable reversible attachment involves cells attaching to a surface through a single pole and frequently returning to the surrounding medium [31]. It can be observed that some species have variations in their numbers during that time. The example is 12 vs. 24 h shown in Table 4 for *M. glauca* and *Aphanocapsa* sp. Overall, these fluctuations did not significantly affect the material performance of the activated carbon.

The variation in cell numbers observed for some cyanobacteria species indicates that the longer adsorption time yielded more favorable results. However, it is evident that the treatment method employed may not have effectively targeted and eliminated certain cyanobacteria cells. It is possible that the structure of material C_AC may not be optimally suited for specific species of cyanobacteria present in the water, thus necessitating further exploration and refinement of the treatment approaches used to address these challenges effectively.

**Figure 3:**
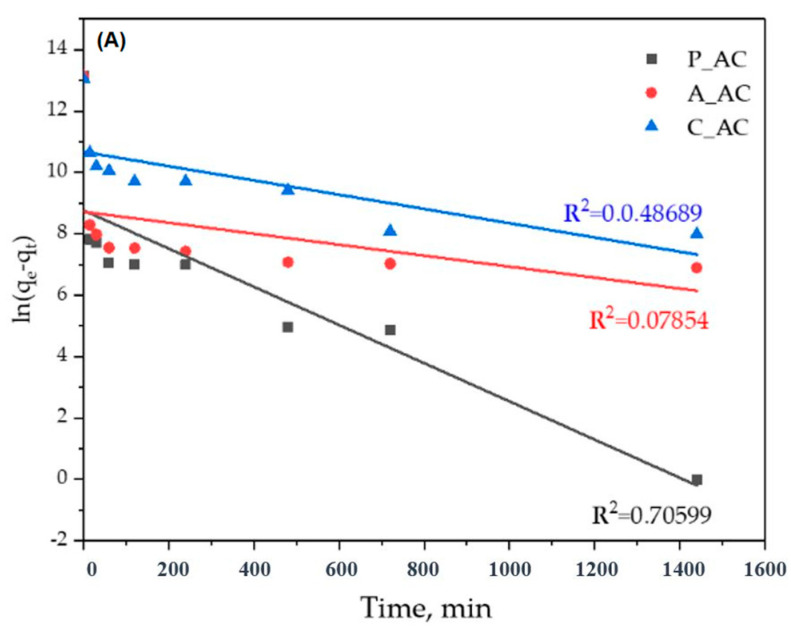

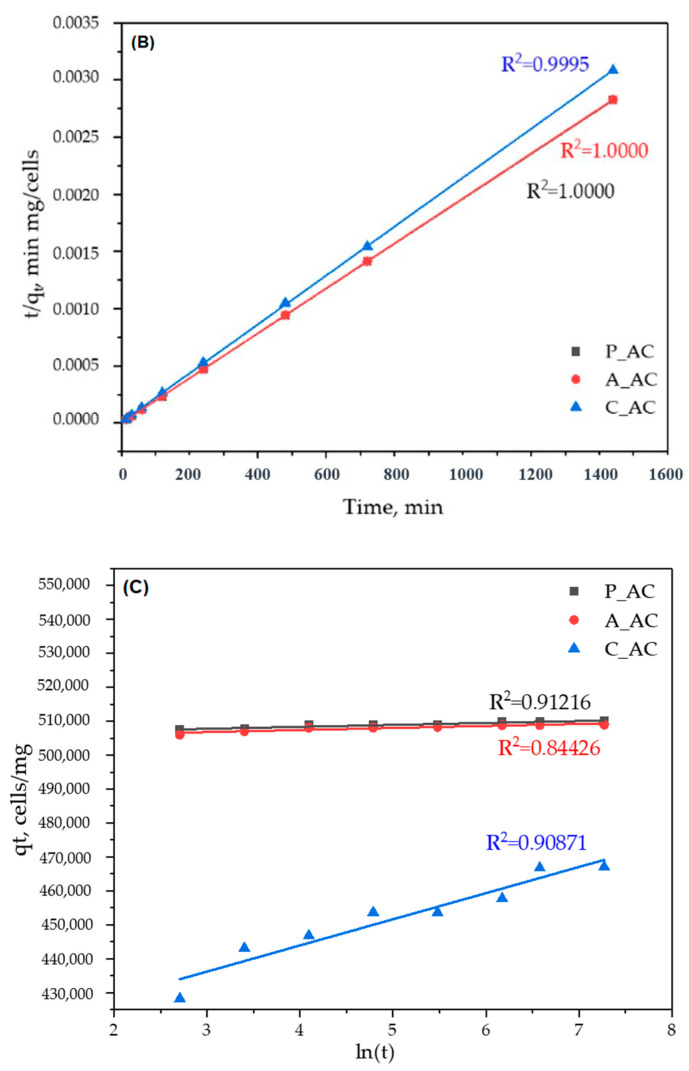
Linear fits of kinetic models: (**A**) pseudo I model; (**B**) pseudo II model; (**C**) Elovich model.

In order to understand kinetic of the adsorption, as well as the cyanobacteria removal mechanism, different kinetic models were used in order to fit the experimental data. For that purpose, pseudo-I, pseudo-II and Elovich models were used, and the results are presented at Figure 3 and Table 5.

As can be seen from the Figure 3 and Table 5, the model that the most relevantly describes the removal of cyanobacteria from contaminated waters by using P_AC, A_AC, and C_AC is the pseudo-II-order model. For this model, the best agreement with the experimental data were obtained for all three adsorbents (R^2^ equal to or approximately equal to 1; very good agreement of the theoretical and experimental q_e_). The rate constants and initial rate constants for all three adsorbents were relatively similar, which means that the removal of cyanobacteria from the water solutions occurred at approximately the same rate. However, if we take into account that approximately the same removal rates were achieved with three materials that differ significantly in terms of the specific surface areas (36.6 for P_AC, 485 for A_AC, and 1100 m^2^/g for C_AC) [15], it can be concluded that the most energetically favorable binding of the cyanobacteria was with the P_AC and then the A_AC, and the least favorable was with the C_AC. 

Good agreement of the experimental results with pseudo-second-order model indicate the following: (1) adsorption occurs at specific sites, where no interaction occurs between the cyanobacteria cells; (2) the adsorption energy is independent of the surface coverage; (3) the attainment of mono-layer coverage on the adsorbent surface yields maximum adsorption; and (4) the amount of cyanobacteria cells does not change [32]. Also, in general, the pseudo-second-order model provides that a complex mechanism is included in the pollutant removal, and the best correlation was observed when the rate-determining step was considered as a chemical reaction between the adsorbent and the adsorbate (chemisorption). In this mechanism, the kinetics of the adsorption process correlate with two competitive reversible second-order reactions at higher adsorbate/adsorbent ratios and a reversible second-order reaction at low adsorbate/adsorbent ratios [32].

However, the experimental results for the P_AC and A_AC show that the state of equilibrium was established very quickly, and more than 99% of the cyanobacteria (about 500,000 cells/mg) were removed already after 15 min (Figure 2). Such a high rate of removal of cyanobacteria indicates that physical adsorption is dominant and that the largest part of the cyanobacteria is removed precisely by a mechanism that does not involve the formation of chemical bonds. On the other hand, the good agreement of the obtained results with the pseudo-II kinetic model may indicate that, although physisorption is dominant, a certain, insignificant part of the cyanobacteria is removed by the chemisorption mechanism. For the C_AC, the equilibrium (maximum removal of cyanobacteria) was achieved after 720 min, which was significantly slower than for the P_AC and A_AC (Figure 2) and can indicate a higher contribution of chemisorption in the cyanobacteria removal in comparison to the P_AC and A_AC.

In addition to good efficiency and quick removal of the pollutants, from the aspect of practical application, it is of particular importance to determine whether the applied materials are ecologically safe, as well as for human health and the environment. For this reason, before adsorption experiments, it was of interest to determine the concentrations of potentially toxic elements, investigate the health risk, and to perform toxicity studies of the materials. Thus, only material(s) that satisfy all the prescribed criteria can be further investigated and potentially applied in practice. 

### 2.3. Concentration of Potentially Toxic Elements (PTEs) in Water after the Treatment

The analyses of *PTE* concentrations in the water sample after contact of 24 h with the P_AC, A_AC, and C_AC are shown in Table 6. In order to compare the obtained values, in Table 6 are also presented the permissible limits of the element concentrations after the materials’ application in drinking water according to the Rulebook on hygienic correctness of drinking water of the Republic of Serbia [27], according to the permissible limits in land and water for irrigation Rulebook on permitted quantities of hazardous and harmful materials in land and water for irrigation and methods of their testing of the Republic of Serbia [28], and according to the permissible limits according to the World Health Organization [29].

The analyses of water samples after the contact with activated carbon materials show that the water samples after the treatment using A_AC and C_AC is suitable for drinking and irrigation according to the Rulebook on hygienic correctness of drinking water of the Republic of Serbia [33], the Rulebook on permitted quantities of hazardous and harmful materials in land and water for irrigation and methods of their testing of the Republic of Serbia [34], and the WHO [35].

Nevertheless, the water sample treated with P_AC indicates that the nickel content approaches the limit set by the regulations outlined in the Republic of Serbia’s rulebooks for drinking water. Additionally, this particular sample exhibited an elevated concentration of molybdenum that surpasses both the drinking water standards defined by the Republic of Serbia’s rulebooks and those set by the World Health Organization. However, to conclude if the material has potential health hazards, they should be estimated through a complete health risk assessment of the total content of potentially toxic elements.

### 2.4. Health Risk Assessment of Heavy Metals

The chronic daily intake (*CDI*) for both the ingestion and dermal contact of water samples treated with activated carbon material was calculated, thereby taking into account the concentrations of the involved elements. The corresponding *CDI* values are presented in Table 7. Additionally, hazard coefficients (*HQ*s) were calculated for all three materials and are shown in Table 8.

**Figure 4:**
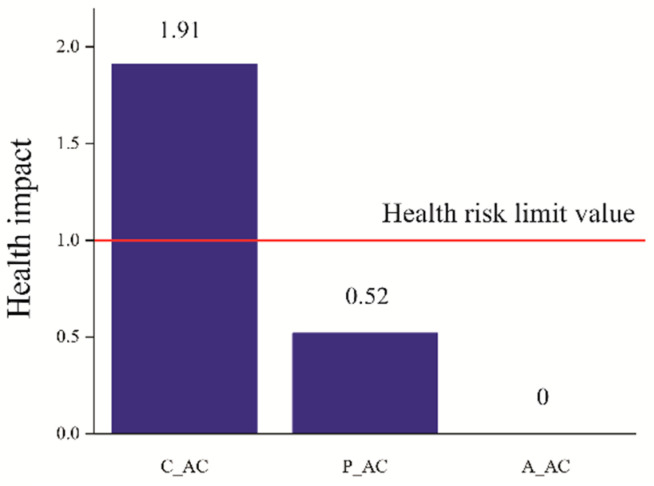
The health impact of C_AC, P_AC, and A_AC, as well as health risk limit value (1).

It is crucial to consider the hazard quotient (*HQ*) to comprehensively assess the potential health effects of the material used for water treatment and of heavy metals in water, thus accounting for both ingestion and dermal exposure pathways. Figure 4 illustrates the health impact (HI) values for the P_AC, A_AC, and C_AC materials, thus providing valuable insights into their overall impact on human health.

Based on the findings depicted in Figure 4, the health index (HI) values for the materials P_AC, A_AC, and C_AC were determined to be 0.52, 0.00, and 1.91, respectively. Consequently, C_AC has been deemed to pose significant risks to human health and should be considered unsafe for use at concentrations of 100 g/L. In the calculation for the P_AC, notable concentrations of Ni (0.021 mg/L) and Cu (0.054 mg/L) were identified. However, the total health impact of those elements did not contribute to the calculated potential adverse health impact. Turning our attention to the A_AC material, it demonstrated no discernible adverse health impact, as the elements examined for its calculations were below the limit of detection. On the other hand, for the C_AC material, elevated concentrations of Cd, Ni, Zn, Cu, and Mn (0.004, 0.008, 0.019, 0.014, and 0.002 mg/L, respectively) were observed, thus collectively contributing to the negative health impact associated with this material.

However, in order to definitively confirm the permanent, safe, and secure use of the tested activated carbons in water purification in terms of their biological impact, it was necessary to conduct cytotoxicity and genotoxicity tests (Section 2.5 below).

### 2.5. Toxicity Studies of Materials

In order to investigate the cytotoxicity of the active carbon materials, an MTT assay was conducted toward normal human fibroblast cells (MRC-5s), as well as alkaline Comet assay for the genotoxicity study.

#### 2.5.1. MTT Assay

**Figure 5:**
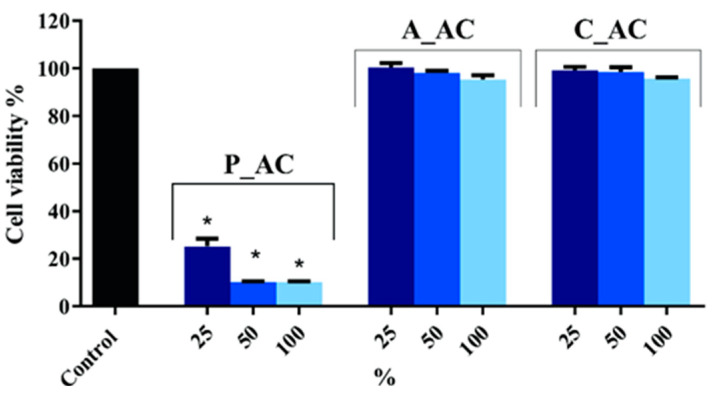
Effects of P_AC, A_AC, and C_AC at different amounts (25, 50, and 100%) on the viability of normal human fibroblast cells (MRC-5s); the level of significance was set to * *p* < 0.05.

The cytotoxicity study was done using an MTT assay on normal human fibroblast cells (MRC-5s) for the materials P_AC, A_AC, and C_AC to determine the cell viability (%) at different concentrations of materials (Figure 5). For investigation, 100 mg of material was used in 2 mL of medium.

Based on the MTT results, it was observed that all the tested amounts of P_AC exhibited significant cytotoxicity towards the MRC-5s. The cell viability for P_AC amounts of 25%, 50%, and 100% was determined to be 25.3%, 10.2%, and 10.1%, respectively. Conversely, the results obtained for the A_AC and C_AC did not indicate any cytotoxicity towards MRC-5s. The cell viabilities for the A_AC were measured at 100.0%, 98.1%, and 95.4% for amounts of 25%, 50%, and 100%, respectively. Similarly, the cell viabilities for C_AC were determined to be 99.2%, 98.6%, and 95.7% for the corresponding amounts of 25%, 50%, and 100%.

The significant cytotoxicity observed for the P_AC suggests that this material is potentially toxic and not safe for further application, although it had a good result in removing cyanobacteria cells. In contrast, the non-cytotoxic effect of the A_AC indicates that these materials, besides having a good result in removing cyanobacteria cells, will not negatively affect human health. Also, C_AC, although it did not show as good results in the removal of cells as A_C, is safe for use in the examined concentration (250 mg in 25 mL). These findings establish a connection between the cytotoxicity of the activated carbon materials and their efficiency in eliminating cyanobacteria cells. This highlights the importance of considering their biological impact on water treatment applications and not only the efficiency in removing the pollutants—in this case, cyanobacteria cells.

#### 2.5.2. Comet Assay

**Figure 6:**
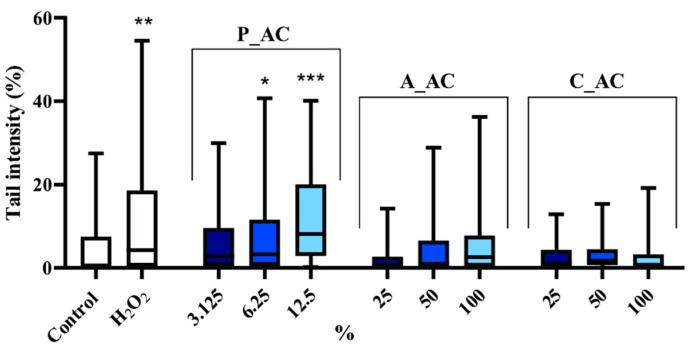
Comet assay for P_AC, A_AC, and C_AC; the level of significance was set to * *p* < 0.05; ** *p* < 0.01; and *** *p* < 0.001.

The study on DNA damage was done using a Comet assay on normal human fibroblast cells (MRC-5s) for P_AC, A_AC, and C_AC to determine the DNA damage at different concentrations of materials (Figure 6). The results of the Comet assay reveal that the P_AC exhibited a strong genotoxic potential, even at low concentrations. Notably, the concentration of 12.5% of the P_AC showed higher genotoxicity compared to the extremely toxic effect induced by H_2_O_2_, which is a known genotoxic agent that severely affects cell proliferation. The presence of nickel in the P_AC is suggests as a possible cause for this toxicity. Nickel has been extensively associated with various negative health impacts, including respiratory diseases, renal disorders, cardiovascular ailments, genotoxic effects, and cancer [36]. These results raise concerns about the suitability of materials derived from P_AC for use when considering their potential genotoxic risks. In contrast, the Comet assay results for the A_AC indicate that the 25% concentration of A_AC exhibited DNA damage levels comparable to those of the control cells, thus suggesting no significant genotoxic effects. However, at 50% and 100% concentrations, the genotoxicity of the A_AC increased, thus surpassing the levels observed in the control cells, but without statistical significance. These findings suggest a concentration-dependent genotoxic response for A_AC. On the other hand, the Comet assay results for the C_AC indicate that all the tested concentrations of C_AC exhibited DNA damage levels below those of the control cells. This suggests that C_AC does not induce significant genotoxic effects under the tested conditions.

The observed genotoxic potential of P_AC raises concerns about its suitability for certain applications, thus considering the potential risks associated with genotoxicity. It suggests that caution should be exercised when using P_AC in contexts where genotoxic effects may pose a significant concern. The concentration-dependent genotoxic response of the A_AC indicates that careful consideration should be given to the concentration used in applications to mitigate potential genotoxic effects. On the other hand, the absence of significant genotoxic effects in the C_AC suggests its potential suitability for applications where genotoxicity is a critical concern. However, it is not a suitable material for the removal of cyanobacteria cells due to poor efficiency. Overall, an understanding the genotoxicity of these materials helps inform their appropriate use and ensures that potential risks are minimized in their application.

Taking into account the non-cytotoxic effect of A_AC and its fairly noticeable genotoxic effect, further research will be dedicated to determining the optimal concentration that ensures both safety and a high degree of cyanobacteria removal from water. This investigation aims to strike the right balance between effectiveness and minimizing potential genotoxic risks. Building upon the positive outcomes of A_AC in cyanobacteria removal, a more detailed study will be conducted to identify the ideal liquid/solid phase ratio for efficient removal while maintaining safety. The initial findings indicate that a concentration of 25 mg of A_AC in 250 mL of water, with an experimental duration of 30 min, shows promising results in terms of cyanobacteria removal. This material will undergo further examination to evaluate its effectiveness in achieving high removal rates while minimizing any potential adverse effects.

### 2.6. Influence of Mass and Volume of the Adsorbent (A_AC) on Cyanobacteria Cell Removal

Based on the cytotoxicity and genotoxicity analysis, the P_AC (material derived from date palm leaf) was found to be undesirable for use due to its high cytotoxic and genotoxic potential. The adverse effects observed in these experiments indicate that using P_AC may pose a risk to both human health and the environment, thus making it unsuitable for further application in the removal of cyanobacteria and cyanotoxins. On the other hand, the effectiveness of the C_AC (commercial activated carbon) in removing cyanobacteria cells did not meet the desired expectations based on preliminary results. As a result, it was decided not to proceed with experiments involving it. 

In the further investigation of the application of active carbon materials, only the material synthesized from the fruit of black alder cone like flowers (A_AC) was selected. The A_AC showed satisfactory results in the initial experiments, especially regarding the removal of cyanobacteria cells. After a 30 min experiment, it showed promising performance in reducing the concentration of cyanobacteria in the water sample. Thus, only the A_AC was considered for further research. The contaminated water purification was carried out in the next year using activated carbon A_AC. The water was taken when the cyanobacterial bloom started again and increased the number of cyanobacteria, specifically in June 2022. In order to ensure consistency and compliance in the subsequent experiments, an adsorption time of 30 min was chosen. That allowed for a systematic assessment of the efficiency of the A_AC in removing cyanobacteria and achieving desired water quality standards. 

The results of examination of the influence of mass of the A_AC (10 mg, 20 mg, 35 mg, 50 mg, 75, mg, 100 mg, 150 mg, 200 mg, and 250 mg) and volume (25 mL, 250 mL, 500 mL, 750 mL, and 1000 mL) on the removal of cyanobacteria cells in contaminated water collected in June 2022 are presented in Table 9, Table 10, Table 11, Table 12 and Table 13.

The results presented in Table 9 showed that for the efficiency of the applied material for cyanobacteria removal higher than 90% for a volume of 25 mL, at least 20 mg of the A_AC was required. For a volume of 250 mL (Table 10) for cyanobacteria removal efficiency higher than 90% at least 75 mg of the A_AC was required, while for a volume of 500 mL (Table 11), at least 50 mg of the A_AC was required in order to obtain efficiency in cyanobacteria removal higher than 90%. For a volume of 750 mL (Table 12), more than 90% of cyanobacteria cells could be removed with at least 200 mg of the A_AC. For a higher reaction volume of 1000 mL (Table 13) for good cyanobacteria removal, (~80%) at least 250 mg of A_AC was required.

**Figure 7:**
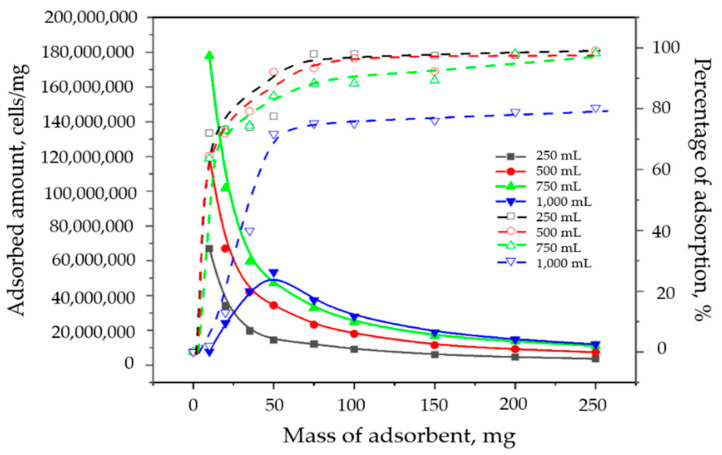
Influence of the mass of the adsorbent on the efficiency of cyanobacteria adsorption (cells/mg) and percentage of the adsorption (dashed line).

From the aspect of the practical application of the material, both from an ecological and economic point of view, in addition to the high efficiency in removing the desired pollutant, it is of particular importance that there is complete predictability in terms of the behavior of the material. For this purpose, in addition to the detailed characterization of the material itself, it is of particular interest to define the method, that is, the mechanism of pollutant removal. In order to achieve that, it is necessary to examine the influence of various parameters and the initial conditions on the efficiency of the material. One of those parameters is the influence of the adsorbent mass on the removal efficiency. The investigations were performed for the mass of adsorbent in the interval from 10 to 250 mg and the reaction volume from 25 to 1000 mL, and the results are shown in Figure 7. 

In Figure 7 are given diagrams of the dependences of the adsorbed amount of the cyanobacteria (cells/mg), as well as the percentage (%) of the mass of the A_AC. As can be seen, increasing the mass of the adsorbent up to 50 mg caused a significant increase in the percentage of the cyanobacteria adsorption (higher than 80% for volumes 250–750 mL and 70% for 1000 mL), which is expected, given that increasing the mass of the adsorbent increases the number of adsorption centers. After that, the percentage of removal increased less (10–20%, up to ~100% for 250–750 mL and up to 80% for 1000 mL), and the system entered equilibrium, so further increasing the amount of adsorbent did not lead to a significant increase in the efficiency percentage. This is because all the amount of cyanobacteria had been removed from the solution (for 250–750 mL), so any further increase in the mass of the adsorbent had no purpose, because the active sites remained unfilled. The establishment of an adsorption equilibrium that was not 100%, which was achieved for the 1000 mL quantity, may be an indication that the molecules of the cyanobacteria partially or completely bound to the surface of the adsorbent in a horizontal position so that one cyanobacterium occupied more than one active sites; thus, an increase in the mass of the adsorbent from 50 to 250 mg was not enough increase of the number of active sites and, consequently, was not sufficient for the adsorption of all cyanobacteria cells.

Also, it is visible from Figure 7 that the best adsorption capacity (cells/mg) for the cyanobacteria removal was obtained for the lowest amount of the A_AC (10 mg in the appropriate volume). The explanation for this phenomenon could be that with a higher content of the solid phase, there is a greater probability of the adsorbent particles’ collision with each other, which can lead to their agglomeration. This causes a decrease in the specific surface area available for cyanobacteria removal and an increase in the length of the diffusion path, which together lead to a decrease in the capacity and efficiency of cyanobacteria removal. The obtained results indicate that by increasing the content of the solid phase, the contact surface between the pollutant and the surface of the adsorbent cannot be increased indefinitely but that even the opposite effect can occur, so special care should be taken when determining the best liquid/solid phase ratio.

In the previous study of Kandić et al. [15], it was shown that the materials were non-porous, mixed, and micro-porous for P_AC, A_AC, and C_AC, respectively. The specific surfaces were 36.6 m^2^/g, 485 m^2^/g, and 1100 m^2^/g for P_AC, A_AC, and C_AC, respectively. To compare the results obtained in our study with the results from different recent studies of cyanobacteria cells removal, the results are summarized in Table 14.

From Table 14, it can be concluded that the removal efficiency for cyanobacteria removal described in the literature is about 90%. The adsorption on material such as polyethyleneimine is described in the papers by Park et al. [14,31]. In the papers, they investigated the removal of *Microcystis aeruginosa* cells on of the representative species of extremely harmful cyanobacteria, which can cause harmful algal blooms. In the paper by Park et al. [17], they investigated a modified chitosan–waste biomass composite fiber, which obtained a removal efficiency of 89.0–91.8%. In the paper by Park et al. [37], two experimental results for the material were shownL on artificial media, where the efficiency was ~90%, and for algal blooming water, which was ~80%. Habtemariam et al. [39] examined the Lake Legedadi Reservoir (Ethiopia) with the dominance of *Microcystis aeruginosa* and *Anabaena* (currently known as *Dolichospermum* spp.). In the study, coagulants of 30 mg/L were used, and a removal efficiency of 93.6%, without causing cell lysis was obtained. Mališová et al. [38] showed the capacity of ferrate in concentrations of up to 90% in water samples from a lake in Šaštín-Gazárka (Slovakia). In this study, it was shown that there was not a large difference in the removal efficiency for concentrations of 5 and 10 mg/L. The most represented cyanobacteria in that lake were *Microcystis wesenbergii*, *Microcystis novacekii*, *Microcystis aeruginosa*, *Microcystis ichthyoblabe*, *Limnothrix redekei*, *Raphidiopsis raciborskii*, *Anabaena cicinalis*, and *Aphanizomenon gracile.* The cyanobacteria community is relatively similar to that in the water from Aleksandrovac Lake, which is the objective of this study. Compared to other studies, the material obtained from black alder cone-like flowers showed higher removal efficiency.

### 2.7. FTIR Analyses of A_AC before and after the Treatment

**Figure 8:**
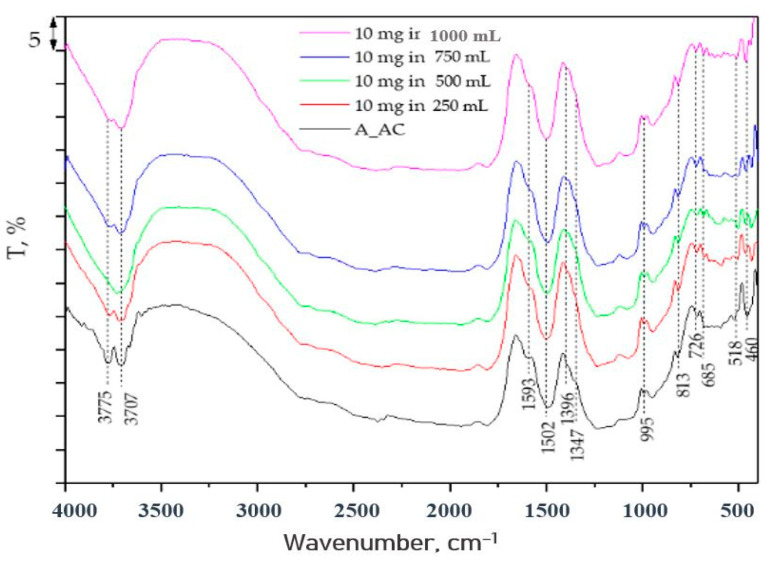
The FTIR spectra of the A_AC before and after adsorption of the cyanobacteria.

In order to further define the potential mechanism of cyanobacteria removal by using A_AC as the adsorbent, the infra-red spectra (FTIR) were recorded for the starting sample (A_AC) and the samples contaminated with cyanobacteria. In order to obtain the best visibility of the process, the spectra were recorded from the previously mentioned experiments, where the lowest amount of A_AC was used (10 mg) for the removal of cyanobacteria from volumes of 250, 500, 750, and 1000 mL, and the results are presented in Figure 8.

As it can be seen in the spectrum of the starting sample for the A_AC, the spectral bands characteristic for cellulose and lignin are visible. The spectral bands at 3775 and 3707 cm^−1^ correspond to O–H stretching vibrations that originated from the physically adsorbed water [40]. The bands in 1600–1500 cm^−1^ are characteristic for aromatic skeletal vibrations of cellulose [41], while small CH bending vibrations can be found in the 1400–1300 cm^−1^ region, and C-O vibrations occur at about 1000 cm^−1^. The band at 995 cm^−1^ also can represent aromatic C-H in plane deformation, while the peak at 813 cm^−1^ represents aromatic C-H out-of-plane vibration [42,43]. The weak signal at 726 cm^−1^ originated due to the presence of a monoclinic cellulose that scribed to a CH_2_ rocking vibration [44]. The band at 685 cm^−1^ originated from a δO-H_oop_ out-of-plane bending mode [44]. The doubled bands at 518 and 460 cm^−1^ can be assigned to C-C vibrations [45].

**Figure 9:**
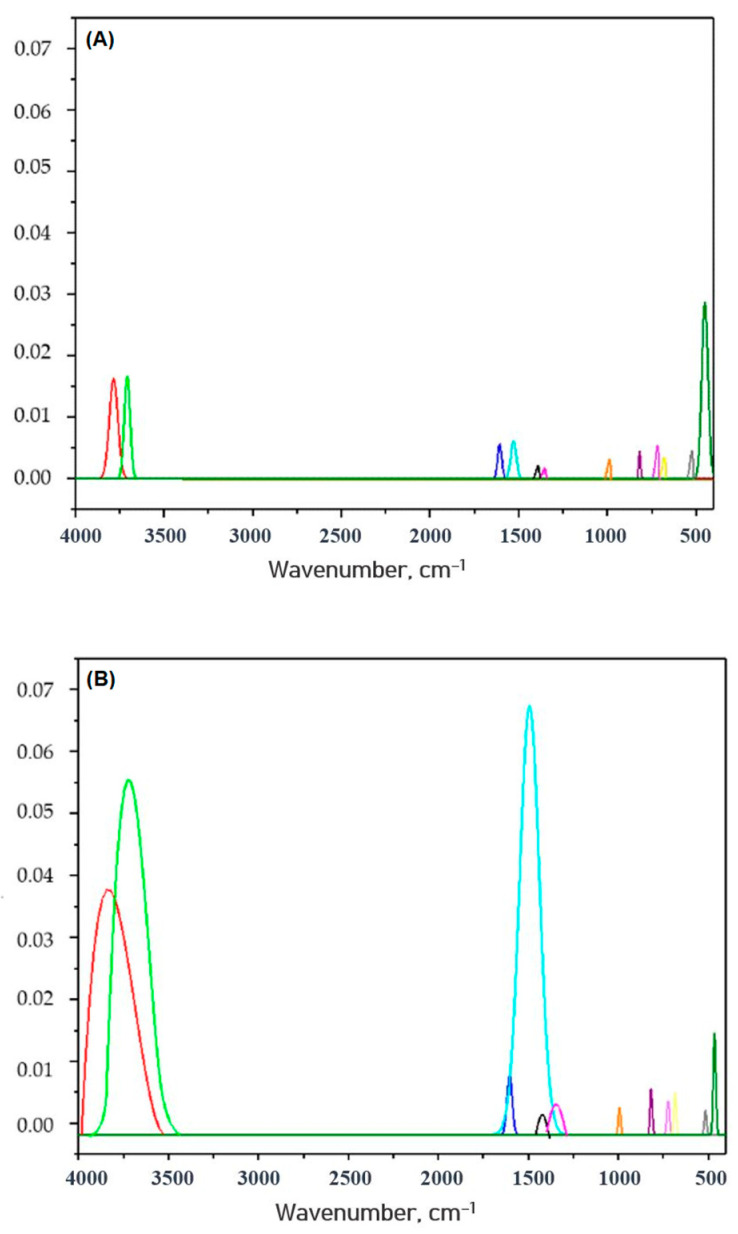
The Gaussian functions for the (**A**) starting A_AC and (**B**) sample after cyanobacteria adsorption.

In the FTIR spectra of the A_AC after cyanobacteria removal, the spectral bands characteristic for the starting sample are also visible, and due to overlapping, bands characteristic for the cyanobacteria, as well as new bands, were not noticed. Thus, in order to find differences, a Gaussian function was used for the deconvolution and fitting of the normalized FTIR spectra. The Gaussian functions for the starting A_AC and sample after cyanobacteria adsorption (10 mg of A_AC in 1000 mL of contaminated water) are given in Figure 9, while the maximum of the spectral bands and the bands areas relative to the area of the band with the lowest area are given in Table 15.

As can be seen from Figure 9 and Table 15, only changes in the intensities of the bands are visible. The most significant increase in the intensities, as well as the peak areas, are notices for peaks where overlapping of the most intense spectral bands occur (in ranges of 3700–3800 cm^−1^ and 1500–1600 cm^−1^). For peaks in the range of 500–1500 cm^−1^, no significant changes were noticed (changes in the peaks areas were below two times), and for the band at 460 cm^−1^, due to presence of cyanobacteria at the surface of the A_AC sample, C-C vibrations had been much more difficult, and as a consequence, the peak intensity and peak area decreased. In the spectrum of the contaminated sample, there were not observed any new spectral bands, which means that no new chemical bonds were formed at the surface of the A_AC sample after cyanobacteria removal or that the number of those bonds was very small. From that point, it can be assumed that physical adsorption occurred at the surface of the A_AC as a dominant process.

It is well known that cellulose and lignin, as the main constituents of the A_AC, possess both polar and non-polar sides [47,48]. For cellulose, its polar character originates from the -OH groups, while the non-polar side originates from the C-H chain [47]. In the lignin, the polar character of molecule is dominant in comparison to the non-polar character due to the presence of hydroxyl groups and benzene rings, where hydroxyl groups have a dominant influence over the other functional groups.

For a cyanobacteria, is known that its cellular wall is mainly constituted from protein molecules—so-called S-layers—and different carbohydrate structures, depending on the kind of cyanobacteria. S-layers are two-dimensional crystalline arrays formed by a single species of (glyco)protein, which covers the entire surface of a cell [49]. One of the most important properties of a protein is the interaction of its polar and non-polar side chains with the environment. The non-polar (water hating) side chains tend to push themselves to the inside of a protein, while the polar (water loving) side chains tend to place themselves to the outside of the molecule [50]. From that reason, the surface of the cyanobacteria also showed a polar character. Finally, it can be assumed that physical adsorption includes weak electrostatic interaction, Van der Waals, and London forces between the -OH groups from the surface of the A_AC and amino acid side chains of the (glyco)proteins. Considering that such interactions do not involve the formation of chemical bonds, they can be the reason and explanation for the very fast kinetics of the cyanobacteria removal. Physisorption is also important for the potential reuse of once-used material, because it enables relatively simple desorption.

## 3. Conclusions

In this study, cyanobacteria removal from contaminated water taken from the eutrophic Aleksandrovac Lake in Serbia was successfully achieved. As adsorbents, activated carbon materials derived from date palm leaf stalk (P_AC), black alder cone-like flowers (A_AC), and commercial activated carbon obtained from coconut shell (C_AC) were used. The influence of the contact time, reaction volume, and mass of the adsorbent, as well as FTIR analysis of the adsorbents before and after cyanobacteria removal, were investigated. Also, the safety of the applied materials was checked, and the determination of the concentrations of potentially toxic elements, investigation of the health risk, and the toxicity of materials were studied. The obtained results can be summarized as follows:

1. The kinetic results showed better removal of cyanobacteria efficiency with the P_AC and A_AC (>99% after 15 min) in comparison to the C_AC (>84% after 15 min and >90% after 12 h). The fast kinetics and good correlation of the results with the pseudo-second-order model indicate the complex mechanism of cyanobacteria removal, which includes physisorption as the dominant phenomena, as well as chemisorption.
2. After treatment with the A_AC and C_AC, the water quality met the Serbian and WHO standards for drinking and irrigation in terms of cyanobacteria removal. However, the P_AC treatment resulted in Ni concentrations that were measured at the Serbian drinking water limit and Mo concentrations that exceeded both the Serbian and WHO limits. Overall, P_AC and A_AC pose no health risk, while C_AC indicates a potential risk to human health.
3. The health risk assessment results for the heavy metals indicate that the health impacts (HIs) for P_AC and A_AC are below the level of 1. However, the P_AC exhibited significant cytotoxicity and strong genotoxic potential toward MRC-5s, while the A_AC and C_AC did not show any cytotoxicity. The A_AC showed a concentration-dependent genotoxic response, while the C_AC did not induce significant genotoxic effects.
4. The results of the influence of the mass of the A_AC on the cyanobacteria removal rate using different reaction volumes showed that for an efficiency higher than 90% for volumes of 25, 250, 500, and 750 mL, at least 20, 75, 50, and 200 mg of A_AC, respectively, are required.
5. FTIR analysis only showed intensity changes in the bands, thus indicating that no new chemical bonds formed and confirming the results of kinetic experiments and that physical adsorption was a dominant process.

Finally, the achieved results give a clear possibility of directing further research on the utilization of activated carbon from black alder cone-like flowers to develop the most effective solution for the global problem of cyanobacteria blooms. 

## 4. Materials and Methods

### 4.1. Materials

In this study, activated carbon materials obtained from waste biomass as the pre-cursor date palm leaf stalk (*Phoenix dactylifera* L.) and black alder cone-like flowers (*Alnus glutinosa* L.) were used. The precursors were carbonized in an atmosphere of N_2_ at 750 °C in the horizontal furnace, and then the activation process was performed in another horizontal furnace in a N_2_-CO_2_-N_2_ atmosphere at 750 °C. The new activated carbons were labelled P_AC for date palm leaf stalk and A_AC for black alder cone-like flowers. The detailed processes and characterization of both activated carbon materials were described in a previous paper: Kandić et al. [15]. For comparative purposes, we also included a commercially available activated carbon material made from coconut shell—AquaSorb^®^ HSL (8 × 30 MESH) (C_AC) was also tested.

### 4.2. Qualitative and Quantitative Phytoplankton Analyses of Aleksandrovac Lake

Water samples for evaluating cyanobacteria cell removal were collected from the Aleksandrovac Lake, which is a highly eutrophic lake situated in the southern region of Serbia, in August 2021 and June 2022. The samples were retrieved from a depth of 1 m to ensure representative analysis. Ruttner’s hydrobiological probe was utilized to obtain the water samples, thus minimizing the risk of contamination from other layers. For qualitative analysis of phytoplankton, a planktonic mesh measuring 25 cm in diameter, with a mesh size of 22 μm, was used to extract samples from the lake bottom to the water surface.

Immediately after collection, all samples were transported to the laboratory and processed within a short time frame of 24 h. They were promptly fixed in 4% formaldehyde. Quantitative analysis was performed using a Zeiss Axio Observer.Z1 inverted light microscope, thus following the standard method (SRPS EN 15204: 2008) established by Utermöhl [51]. Qualitative phytoplankton analysis involved microscopic examination of the water samples and identification of observed taxa. 

For species identification, standard keys provided by Huber-Pestalozzi et al. [52], Komárek et al. [53,54,55], Krammer et al. [56,57], Starmach [58], and John et al. [59] were utilized. The classification system proposed by Reynolds [60] was employed to categorize species into divisions, including Cyanobacteria, Chlorophyta, Euglenophyta, Cryptophyta, Xanthophyta, Chrysophyta, Bacillariophyta, and Dinophyta.

### 4.3. Influence of Contact Time on the Cyanobacteria Cells Removal

The activated carbon materials (250 mg) were mixed with 25 mL of contaminated water and placed on a shaker at 110 rpm for different time periods (15 min–24 h) at a temperature of 25 °C. The water used for the experiment was collected in August 2021. After a specific time, the suspensions were rapidly filtered through filter paper using a vacuum pump (filtering time was less than 15 s). Subsequently, the number of cyanobacteria cells present in the filtrate was determined.

The removal efficiency (*RE*) of activated carbon (%) is estimated by the following equation:

(1) $$RE=\frac{\left(C_{i}-C_{t}\right)}{C_{i}}\times 100,$$

where *C_i_* (cells/mL) is the initial concentration of cyanobacteria cells, and *C_t_* (cells/mL) is the concentration of cyanobacteria cells at time *t* [61].

The experimental results were further analyzed using different kinetic models in order to explain the kinetics of the cyanobacteria cell removal. For that purpose, three kinetic models were used: Pseudo-first, pseudo-second, and Elovich model. The applied kinetic models are given in Table S1 [62]. 

### 4.4. Concentration of Potentially Toxic Elements (PTEs) in Water after the Treatment

To determine potentially toxic element (PTE) amounts, 250 mg of each sample was taken and put in a closed PFA digestion vessel, and then 7 mL of 70% nitric acid (Macron Fine Chemicals, Gliwice, Polska) and 1 mL of 30% hydrogen peroxide (Sigma-Aldrich, St. Louis, MO, USA) were added. The samples were then digested in a Jupiter-A microwave oven according to the following procedure: during 10 min, the temperature raised up to 150 °C and after that during further for 20 min up to 190 °C, where it was held for 10 min. Each sample was prepared in triplicate, and a blank was prepared in order to discriminate possible impurities. After digestion, the vessels were cooled down to room temperature, transferred to volumetric flasks, and diluted in 20 mL of deionized water. All solutions were stored in polyethylene flasks until the measurements of PTEs were conducted using inductively coupled plasma–optical emission spectrometry (ICP-OES) using a iCAP™ 7400 ICP-OES analyzer (Thermo Scientific, Waltham, MA USA). The single element mercury (Hg) 1000 ppm calibration standard obtained from J.T. Baker and the Multi-element ICP IV 1000 ppm standard obtained from AccuStandard were used to prepare the set of calibration standards for the ICP-OES analysis. All measurements were repeated three times. 

### 4.5. Health Risk Assessment of Heavy Metals

The estimation of chronic daily intake (*CDI*) for health risk assessment involves considering various pathways, including ingestion and dermal adsorption routes, as well as hazard quotient (*HQ*). These parameters for health risk assessment are established by the United States Environmental Protection Agency [63] and the United States Environmental Protection Agency’s Integrated Risk Information System [64]. Table S2 [63,65,66,67,68,69] and Table S3 [64] present the parameters used in the equations for *CDI* and *HQ*. The values are calculated based on the following equations:

(2) $${CDI}_{ing}=\frac{\left(C_{w}\cdot IR\cdot EF\cdot ED\right)}{\left(BW\cdot AT\right)},$$

(3) $${CDI}_{derm}=\frac{\left(C_{w}\cdot SA\cdot K_{p}\cdot CF\cdot ET\cdot EF\cdot ED\right)}{\left(BW\cdot AT\right)},$$

(4) $$HQ=\frac{CDI}{RfD},$$

The complete health impact is a summary of the hazard quotient of ingestion and dermal adsorption.

(5) $$HI=\sum {HQ}_{ing}+\sum {HQ}_{derm}$$

If the value of *HI* is less than 1, there is no health risk, and if the value is equal to or above 1, there may be a concern for negative effects on human health.

### 4.6. Toxicity Studies of Materials

#### 4.6.1. Cytotoxicity Assay

The cytotoxicity was assessed using the MTT (3, (4,5-dimethylthiazol-2-yl)2,5-diphenyltetrazolium bromide) reduction assay, which is a widely recognized method. To determine cell viability, the normal human fetal fibroblast cells (MRC-5s) cell line was employed. The experimental procedure followed the methodology outlined by Mosmann in 1983 [70], where absorbance at 570 nm was measured to evaluate cell viability. The cytotoxicity assessment was conducted over a 24 h period. Initially, the material concentration was 100 mg in 2 mL of medium, and cell viability was measured at 100%, 50%, and 25%. The MTT assay results were obtained using a MULTISCAN reader, thus employing spectrophotometric analysis.

#### 4.6.2. Genotoxicity Assay

The genotoxic potential of activated carbon materials was investigated using the alkaline Comet assay in accordance with MIRCA [71] recommendations, which constitute a highly sensitive method for evaluating DNA damage. The MRC-5s cell line was exposed to the materials for a duration of 24 h. The initial concentration of the materials in the medium was set at 100 mg in 2 mL. The assay encompassed a range of concentrations: 12.5%, 6.25%, and 3.125% for P_AC and 100%, 50%, and 25% for both A_AC and C_AC. The extent of DNA damage was quantified using advanced Comet Assay IV software, thus enabling accurate and precise analysis. The measurement of DNA damage was presented as tail intensity, i.e., the percentage of DNA in the tail of comets.

### 4.7. Influence of Mass of the Adsorbent (A_AC) and Volume of Contaminated Water on Cyanobacteria Cells Removal

After the completion of the first series of experiments, it was determined that activated carbon P_AC showed toxicity (results present in Section 2.5), while C_AC had a low efficiency in removing cyanobacteria (results present in Section 2.2). In accordance with these results, the second series of research continued on contaminated water taken in next year, and included the application of only activated carbon A_AC, when the cyanobacterial bloom started again with increases in the number of cyanobacteria, specifically in June 2022. The experiments were conducted under specific conditions as outlined below: The activated carbons were utilized in varying amounts of 10 mg, 20 mg, 35 mg, 50 mg, 75 mg, 100 mg, 150 mg, 200 mg, and 250 mg. The volumes of the water samples were 25 mL, 250 mL, 500 mL, 750 mL, and 1000 mL (for water samples collected in June 2022). The samples were subjected to stirring in a shaker for 30 min at 110 rpm and temperature of 25 °C. After adsorption time, the suspensions were filtered through filter paper using a vacuum pump (filtering process took less than 15 s). Subsequently, the number of cyanobacteria present in the filtrate was determined.

### 4.8. Fourier Transform Infra-Red (FTIR) Spectra of A_AC before and after the Water Treatment

The activated carbon A_AC samples before and after water treatment were analyzed using a Thermo Fisher Scientific FTIR spectrometer IS-50 in the transmission mode to obtain Fourier transform infra-red (FTIR) spectra. To prepare the samples, a technique involving pressed KBr pellets was employed, where 0.2 mg of the sample was combined with 80 mg of KBr. The spectra were recorded within the range from 4000 to 400 cm^−1^ utilizing 64 scans and a resolution of 2 cm^−1^. Following the recording of the spectra, two corrections were implemented: automatic baseline correction and atmospheric suppression, thus ensuring accurate and reliable results.

### 4.9. Statistical Analysis

Results obtained from MTT assay were analyzed using GraphPad Prism 6.01 Software (Software, Inc., Boston, MA, USA) and by applying one-way ANOVA with Dunnet’s post hoc tests; the level of significance was set to * *p* < 0.05. Data obtained in the alkaline comet assay were analyzed using Statistica 7.0 Software (StatSoft, Inc., Tulsa, OK, USA) by applying non-parametric Mann–Whitney U test. The level of significance was set to * *p* < 0.05; ** *p* < 0.01; and *** *p* < 0.001.

## Figures and Tables

**Table 1:** Cyanobacteria cell number in August 2021 [15] and June 2022 (this study) in Aleksandrovac Lake.

Cyanobacteria	August 2021	June 2022
	[Cell/mL]	
*Anabaenopsis elenkinii*	280	0
*Anathece minutissima*	1280	28,821
*Aphanocapsa Nägeli* sp.	640	56,172
*Glaucospira* sp.	49,150	37,615
*Jaaginema subtilissimum*	1,050,812	0
*Limnothrix planctonica*	1,018,116	321,233
*Merismopedia glauca*	842,052	0
*Microcystyis aeruginosa*	63,193	14,043
*Microcystis flos-aquae*	17,554	27,351
*Oscillatoria limosa*	14,043	0
*Planktolyngbya limnetica*	129,898	0
*Pseudanabaena limnetica*	59,683	172,026
*Rhapidiopsis raciborskii*	1,330,286	2,752,423
*Snowella* sp.	484,483	315,967
*Synechocystis aquatilis*	31,597	0
**SUM**	**5,093,067**	**3,725,651**

**Table 2:** Cyanobacteria cell number before and after water treatment using P_AC (250 mg/25 mL).

Cyanobacteria	Before Treatment	After Treatment with P_AC							
		15 min	30 min	1 h	2 h	4 h	8 h	12 h	24 h
*A. elenkinii*	280	0	0	0	0	0	0	0	0
*A. minutissima*	1280	0	0	0	0	0	0	0	0
*Aphanocapsa* sp.	640	411	183	137	112	32	0	0	0
*Glaucospira* sp.	49,150	4900	4702	3792	442	767	670	308	143
*J. subtilissimum*	1,050,812	3792	2719	2077	1260	235	392	12	0
*L. planctonica*	1,018,116	4279	4450	4010	1604	1666	942	892	463
*M. glauca*	842,052	0	0	0	0	0	0	0	0
*M. aeruginosa*	63,193	0	0	0	0	0	0	0	0
*M. flos-aquae*	17,554	0	0	0	0	0	0	0	0
*O. limosa*	14,043	0	0	0	0	0	0	0	0
*Pl. limnetica*	129,898	0	0	0	0	0	0	0	0
*Ps. limnetica*	59,683	2112	1918	448	280	672	0	0	0
*R. raciborskii*	133,0286	1962	1594	1202	386	533	494	154	104
*Snowella* sp.	484,483	466	206	103	0	0	0	0	0
*S. aquatilis*	31,597	0	0	0	0	0	0	0	0
*SUM*	5,093,067	17,922	15,772	11,769	4084	3905	2498	1366	710
**Removal efficiency (%)**		**99.65**	**99.69**	**99.77**	**99.92**	**99.92**	**99.95**	**99.97**	**99.99**

**Table 3:** Cyanobacteria cell number before and after water treatment by A_AC (250 mg/25 mL).

Cyanobacteria	Before Treatment	After Treatment with P_AC							
		15 min	30 min	1 h	2 h	4 h	8 h	12 h	24 h
*A. elenkinii*	280	0	0	0	0	0	0	0	0
*A. minutissima*	1280	0	0	0	0	0	0	0	0
*Aphanocapsa* sp.	640	0	0	0	0	0	0	0	0
*Glaucospira* sp.	49,150	4465	2444	2250	2996	539	940	499	93
*J. subtilissimum*	1,050,812	2454	4049	2732	2288	1976	933	872	193
*L. planctonica*	1,018,116	3974	3153	3098	3111	1110	734	712	194
*M. glauca*	842,052	137	0	0	0	0	0	0	0
*M. aeruginosa*	63,193	0	0	0	0	0	0	0	0
*M. flos-aquae*	17,554	0	0	0	0	0	0	0	0
*O. limosa*	14,043	9288	5024	0	0	0	0	0	0
*Pl. limnetica*	129,898	0	0	0	0	0	0	0	0
*Ps. limnetica*	59,683	320	242	189	112	68	74	84	28
*R. raciborskii*	133,0286	3616	941	669	229	203	213	194	98
*Snowella* sp.	484,483	2674	571	221	82	48	40	0	0
*S. aquatilis*	31,597	0	0	0	0	0	0	0	0
*SUM*	5,093,067	26,928	16,424	9159	8818	3944	2934	2361	606
**Removal efficiency (%)**		**99.47**	**99.68**	**99.82**	**99.83**	**99.92**	**99.94**	**99.95**	**99.99**

**Table 4:** Cyanobacteria cell number before and after water treatment by C_AC (250 mg/25 mL).

Cyanobacteria	Before Treatment	After Treatment with C_AC							
		15 min	30 min	1 h	2 h	4 h	8 h	12 h	24 h
*A. elenkinii*	280	0	0	0	0	0	0	0	0
*A. minutissima*	1280	415	297	706	277	356	118	59	0
*Aphanocapsa* sp.	640	556	415	358	289	237	320	190	247
*Glaucospira* sp.	49,150	47,384	48,256	37,589	38,385	29,256	27,384	28,256	20,876
*J. subtilissimum*	1,050,812	194,846	126,898	97,423	64,949	87,423	73,658	75,540	15,825
*L. planctonica*	1,018,116	136,919	104,971	105,368	91,279	69,243	77,587	68,460	6206
*M. glauca*	842,052	271,813	229,510	228,982	226,631	261,208	281,208	224,951	362,880
*M. aeruginosa*	63,193	8912	6812	5955	6975	6912	6971	0	0
*M. flos-aquae*	17,554	16,338	17,941	15,939	18,943	12,561	541	0	0
*O. limosa*	14,043	6684	7280	13,368	11,824	8434	3832	140	0
*Pl. limnetica*	129,898	11,140	10,144	12,680	9283	8270	1280	222	0
*Ps. limnetica*	59,683	47,530	49,419	56,025	59,413	47,463	31,589	10,679	10,584
*R. raciborskii*	1,330,286	29,653	28,587	25,128	21,441	23,810	11,010	16,943	5916
*Snowella* sp.	484,483	29,706	23,765	19,706	2858	478	183	0	0
*S. aquatilis*	31,597	8912	7427	5941	4129	1485	0	0	0
*SUM*	5,093,067	810,808	661,722	625,168	556,676	557,145	515,681	425,440	422,534
**Removal efficiency (%)**		**84.08**	**87.01**	**87.73**	**89.07**	**89.06**	**89.87**	**91.65**	**91.70**

**Table 5:** Characteristic parameters of the kinetics models.

Sample	P_AC	A_AC	C_AC
Kinetic model	Pseudo I order model		
R^2^	0.70599	0.07854	0.48689
Kinetic model	Elovich model		
R^2^	0.91216	0.84426	0.90871
Kinetic model	Pseudo II order model		
q_e, experimental,_ cells/mg	509,999	509,009	467,053
q_e,theoretic_ (cells/mg)	510,058	509,048	467,864
k_2,_ mg min/cells	3.84 × 10^−12^	3.86 × 10^−12^	4.57 × 10^−12^
h, cell min/mg	0.99901	0.99765	1.00036
R^2^	1.0000	1.0000	0.9995

**Table 6:** Element concentrations in water solutions before materials’ application and their permissible limits in water.

Element	Material			Permissible Limits		
	P_AC	A_AC	C_AC	[33]	[34]	[35]
	[mg/L]					
Cd	<0.001	<0.001 ^a^	0.004 ± 0.001	0.003	0.01	0.003
Co	<0.001 ^a^	<0.001 ^a^	<0.001 ^a^	/	/	/
Fe	<0.01	<0.01	<0.01 ^a^	/	/	0.3
Ni	0.021 ± 0.001	<0.003	0.008 ± 0.001	0.02	0.1	0.07
Pb	<0.01 ^a^	<0.01 ^a^	<0.01 ^a^	0.01	0.1	0.01
Hg	<0.001 ^a^	<0.001 ^a^	<0.001 ^a^	0.001	0.001	0.006
Be	<0.001 ^a^	<0.001 ^a^	<0.001 ^a^	/	/	/
Zn	<0.001 ^a^	<0.001 ^a^	0.019 ± 0.002	3.0	1.0	0.05
Cu	0.054 ± 0.014	<0.001 ^a^	0.014 ± 0.002	2.0	0.1	2.0
Mo	0.493 ± 0.015	0.040 ± 0.001	0.004 ± 0.001	0.07	/	0.07
As	<0.003 ^a^	<0.003 ^a^	<0.003 ^a^	0.01	0.05	0.01
V	0.014 ± 0.001	<0.001 ^a^	<0.001 ^a^	/	/	/
Sb	<0.003 ^a^	<0.003 ^a^	<0.003 ^a^	0.003	/	0.005
Ti	<0.001 ^a^	<0.001 ^a^	<0.001 ^a^	/	/	/
Se	<0.003 ^a^	<0.003 ^a^	<0.003 ^a^	0.01		0.04
Sr	0.025 ± 0.005	0.009 ± 0.003	0.013 ± 0.002	/	/	/
Ca	1.08 ± 0.10	0.90 ± 0.08	1.30 ± 0.05	200.0	/	100
Al	<0.03	<0.03	<0.03	/	/	/
B	0.70 ± 0.07	0.12 ± 0.01	0.08 ± 0.01	1.0	1.0	2.4
Cr	<0.001 ^a^	<0.001 ^a^	<0.001 ^a^	0.05	0.5	0.05
Mg	0.76 ± 0.10	0.45 ± 0.10	0.40 ± 0.05	50.0	/	150
Mn	<0.001 ^a^	<0.001 ^a^	0.002 ± 0.001	0.05	/	0.5
Ag	<0.03 ^a^	<0.03 ^a^	<0.03 ^a^	/	/	0.005
Sn	<0.004 ^a^	<0.004 ^a^	<0.004 ^a^	/	/	/

^a^ Concentration values below the detection limits.

**Table 7:** The heavy metals of chronic daily intake (*CDI*) through different pathways.

Element	*CDI* Ingestion			*CDI* Dermal		
	P_AC	A_AC	C_AC	P_AC	A_AC	C_AC
Cd	0	0	0.00014	0	0	0.00072
Fe	0	0	0	0	0	0
Ni	0.00072	0	0.00027	0.00379	0	0.00144
Pb	0	0	0	0	0	0
Hg	0	0	0	0	0	0
Zn	0	0	0.000651	0	0	0.00343
Cu	0.00185	0	0.00048	0.00975	0	0.00253
As	0	0	0	0	0	0
Cr	0	0	0	0	0	0
Mn	0	0	0.000068	0	0	0.000361

**Table 8:** Hazard quotient (*HQ*) of heavy metals in water through ingestion and dermal pathway.

Element	*HQ* Ingestion			*HQ* Dermal		
	P_AC	A_AC	C_AC	P_AC	A_AC	C_AC
Cd	0	0	0.274	0	0	1.44
Fe	0	0	0	0	0	0
Ni	0.0360	0	0.0137	0.190	0	0.0722
Pb	0	0	0	0	0	0
Hg	0	0	0	0	0	0
Zn	0	0	0.00217	0	0	0.0144
Cu	0.0462	0	0.0120	0.244	0	0.0632
As	0	0	0	0	0	0
Cr	0	0	0	0	0	0
Mn	0	0	0.00285	0	0	0.015

**Table 9:** Effect of A_AC mass on cyanobacteria cell removal. Reaction volume of 25 mL.

Cyanobacteria	Before Treatment		After Treatment with A_AC [mg]							
		10	20	35	50	75	100	150	200	250
		[Cell/mL]								
*A. elenkinii*	0	0	0	0	0	0	0	0	0	0
*A. minutissima*	28,821	4686	2023	1372	1546	678	17	13	0	0
*Aphanocapsa* sp.	56,172	8429	46,15	2498	2911	1450	30	35	0	0
*Glaucospira* sp.	37,615	5644	3091	1674	1949	971	20	23	0	0
*J. subtilissimum*	0	0	0	0	0	0	0	0	0	0
*L. planctonica*	321,233	48,201	26,393	14,287	16,645	8295	173	189	0	0
*M. glauca*	0	0	0	0	0	0	0	0	0	0
*M. aeruginosa*	14,043	2107	1154	625	728	365	8	0	0	0
*M. flos-aquae*	27,351	3742	2593	1126	1364	772	13	21	0	0
*O. limosa*	0	0	0	0	0	0	0	0	0	0
*Pl. limnetica*	0	0	0	0	0	0	0	0	0	0
*Ps. limnetica*	172,026	25,813	14,134	7651	8914	4442	93	106	0	0
*R. raciborskii*	2,752,423	413,000	226,147	122,417	142,623	71,073	1483	1695	0	0
*Snowella* sp.	315,967	47,411	25,961	14,053	16,373	8159	171	195	0	0
*S. aquatilis*	0	0	0	0	0	0	0	0	0	0
*SUM*	3,725,651	559,033	306,111	165,703	193,053	96,205	2008	2295	0	0
**Removal efficiency (%)**		**85.00**	**91.78**	**95.55**	**94.82**	**97.42**	**99.95**	**99.94**	**100**	**100**

**Table 10:** Effect of A_AC mass on cyanobacteria cell removal. Reaction volume of 250 mL.

Cyanobacteria	Before Treatment		After Treatment with A_AC [mg]							
		10	20	35	50	75	100	150	200	250
		[Cell/mL]								
*A. elenkinii*	0	0	0	0	0	0	0	0	0	0
*A. minutissima*	28,821	0	0	0	0	0	0	0	0	0
*Aphanocapsa* sp.	56,172	0	0	0	0	0	0	0	0	0
*Glaucospira* sp.	37,615	122,876	215,033	134,188	107,955	24,296	26,488	31,934	13,615	28,015
*J. subtilissimum*	0	0	0	0	0	0	0	0	0	0
*L. planctonica*	321,233	289,792	162,758	223,283	143,063	11,495	7569	5421	7625	5050
*M. glauca*	0	0	0	0	0	0	0	0	0	0
*M. aeruginosa*	14,043	0	0	0	0	0	0	0	0	0
*M. flos-aquae*	27,351	0	0	0	0	0	0	0	0	0
*O. limosa*	0	0	0	0	0	0	0	0	0	0
*Pl. limnetica*	0	0	0	0	0	0	0	0	0	0
*Ps. limnetica*	172,026	98,582	109,418	49,853	82,854	9803	7135	0	0	0
*R. raciborskii*	2,752,423	531,878	511,164	569,381	501,598	31,404	32,253	56,146	70,552	21,337
*Snowella* sp.	315,967	0	0	0	0	0	0	0	0	0
*S. aquatilis*	0	0	0	0	0	0	0	0	0	0
*SUM*	3,725,651	1,043,128	998,374	976,706	835,469	76,998	73,445	93,502	91,792	54,401
**Removal efficiency (%)**		**72.00**	**73.20**	**73.78**	**77.57**	**97.93**	**98.03**	**97.49**	**97.54**	**98.54**

**Table 11:** Effect of A_AC mass on cyanobacteria cell removal. Reaction volume of 500 mL.

Cyanobacteria	Before Treatment		After Treatment with A_AC [mg]							
		10	20	35	50	75	100	150	200	250
		[Cell/mL]								
*A. elenkinii*	0	0	0	0	0	0	0	0	0	0
*A. minutissima*	28,821	0	0	0	0	0	0	0	0	0
*Aphanocapsa* sp.	56,172	0	0	0	0	0	0	0	0	0
*Glaucospira* sp.	37,615	165,395	174,074	108,833	40,537	50,129	37,298	27,973	25,652	9324
*J. subtilissimum*	0	0	0	0	0	0	0	0	0	0
*L. planctonica*	321,233	145,345	291,567	166,760	53,843	50,395	14,333	12,110	11,467	2867
*M. glauca*	0	0	0	0	0	0	0	0	0	0
*M. aeruginosa*	14,043	0	0	0	0	0	0	0	0	0
*M. flos-aquae*	27,351	0	0	0	0	0	0	0	0	0
*O. limosa*	0	0	0	0	0	0	0	0	0	0
*Pl. limnetica*	0	0	0	0	0	0	0	0	0	0
*Ps. limnetica*	172,026	52,924	82,502	39,788	27,381	14,927	14,185	0	0	0
*R. raciborskii*	2,752,423	961,693	500,388	462,749	175,320	133,115	63,789	248,845	46,456	23,871
*Snowella* sp.	315,967	0	0	0	0	0	0	0	0	0
*S. aquatilis*	0	0	0	0	0	0	0	0	0	0
*SUM*	3,725,651	1,325,357	1,048,533	778,131	297,081	248,566	129,605	288,918	83,575	36,062
**Removal efficiency (%)**		**64.43**	**71.86**	**79.11**	**92.03**	**93.33**	**96.52**	**92.25**	**97.76**	**99.03**

**Table 12:** Effect of A_AC mass on cyanobacteria cell removal. Reaction volume of 750 mL.

Cyanobacteria	Before Treatment		After Treatment with A_AC [mg]							
		10	20	35	50	75	100	150	200	250
		[Cell/mL]								
*A. elenkinii*	0	0	0	0	0	0	0	0	0	0
*A. minutissima*	28,821	0	0	0	0	0	0	0	0	0
*Aphanocapsa* sp.	56,172	0	0	0	0	0	0	0	0	0
*Glaucospira* sp.	37,615	205,964	129,898	44,079	138,089	76,066	98,301	133,408	19,755	25,300
*J. subtilissimum*	0	0	0	0	0	0	0	0	0	0
*L. planctonica*	321,233	296,052	181,757	141,834	125,334	130,951	97,047	115,476	15,013	9751
*M. glauca*	0	0	0	0	0	0	0	0	0	0
*M. aeruginosa*	14,043	0	0	0	0	0	0	0	0	0
*M. flos-aquae*	27,351	0	0	0	0	0	0	0	0	0
*O. limosa*	0	0	0	0	0	0	0	0	0	0
*Pl. limnetica*	0	0	0	0	0	0	0	0	0	0
*Ps. limnetica*	172,026	90,226	69,513	43,533	47,044	52,310	24,575	16,528	5124	0
*R. raciborskii*	2,752,423	761,612	627,741	722,210	278,878	175,537	217,165	132,004	32,306	30,436
*Snowella* sp.	315,967	0	0	0	0	0	0	0	0	0
*S. aquatilis*	0	0	0	0	0	0	0	0	0	0
*SUM*	3,725,651	1,353,854	1,008,907	951,657	589,345	434,864	437,088	397,416	72,197	65,487
**Removal efficiency (%)**		**63.66**	**72.92**	**74.46**	**84.18**	**88.33**	**88.27**	**89.33**	**98.06**	**98.24**

**Table 13:** Effect of A_AC mass on cyanobacteria cell removal. Reaction volume of 1000 mL.

Cyanobacteria	Before Treatment		After Treatment with A_AC [mg]							
		10	20	35	50	75	100	150	200	250
		[Cell/mL]								
*A. elenkinii*	0	0	0	0	0	0	0	0	0	0
*A. minutissima*	28,821	28,192	23,705	17,478	7611	7731	6391	7107	5657	5645
*Aphanocapsa* sp.	56,172	54,999	48,887	33,777	15,874	13,960	13,964	13,450	11,858	11,046
*Glaucospira* sp.	37,615	36,829	32,736	22,618	10,630	9348	9351	9007	7941	7397
*J. subtilissimum*	0	0	0	0	0	0	0	0	0	0
*L. planctonica*	321,233	314,528	279,570	193,161	90,781	79,835	79,859	76,918	67,812	63,169
*M. glauca*	0	0	0	0	0	0	0	0	0	0
*M. aeruginosa*	14,043	13,749	12,221	8444	3969	3490	3492	3363	2964	2761
*M. flos-aquae*	27,351	26,807	25,182	16,299	2263	6229	7573	6343	6201	5401
*O. limosa*	0	0	0	0	0	0	0	0	0	0
*Pl. limnetica*	0	0	0	0	0	0	0	0	0	0
*Ps. limnetica*	172,026	168,439	149,715	103,441	48,616	42,753	42,766	41,190	36,315	33,828
*R. raciborskii*	2,752,423	2,694,975	2,395,440	1,655,063	777,841	684,052	684,252	659,056	581,036	541,250
*Snowella* sp.	315,967	309,372	274,987	189,995	89,293	78,528	78,549	75,657	66,701	62,133
*S. aquatilis*	0	0	0	0	0	0	0	0	0	0
*SUM*	3,725,651	3,647,890	3,242,443	2,240,276	1052,878	925,926	926,197	892,091	786,485	732,630
**Removal efficiency (%)**		**2.09**	**12.97**	**39.87**	**71.74**	**75.15**	**75.14**	**76.06**	**78.89**	**80.34**

**Table 14:** Literature data on cyanobacteria cells removal by adsorption and coagulation.

Method	Material	Removal Efficiency		Reference
Adsorption	PEI *—modified chitosan-waste–biomass composite fiber PEI * polyvinyl chloride composite fiber	89.0–91.8%		[17]
		80–90%		[37]
Coagulation	Potassium ferrate	~90%		[38]
	Chitosan	93.6%		[39]
Adsorption	Activated carbon	P_AC	99%	This paper
		A_AC	99%	
		C_AC	91.7%	

* PEI—polyethyleneimine.

**Table 15:** Characteristic parameters of Gaussian function. ${\tilde{\nu }}^{\prime }$—bands characteristic of cyanobacteria [46], $\tilde{\nu }$—band maximum, A—relative band area, Δ—difference between band area of contaminated and starting A_AC.

Starting A_AC Sample			Contaminated A_AC Sample		
${\tilde{\nu }}^{\prime }$, cm^−1^	${\tilde{\nu }}^{\prime }$, cm^−1^	A	$\tilde{\nu }$, cm^−1^	A	Δ
3568	3775	0.98175	3775	15.12997	+14.14822 (+15.4 times)
2925	3707	0.68266	3707	1.78243	+1.09977 (+2.60 times)
1732	1593	0.16457	1593	0.34478	+0.18021 (+2.10 times)
1664	1502	0.23796	1502	9.202262	+8.96430 (+38.7 times)
1543	1396	0.01450	1396	0.0137	−0.0008 (−0.06 times)
1453	1347	0.04556	1347	0.05415	+0.00859 (+1.20 times)
1380	995	0.05281	995	0.07417	+0.02136 (+1.40 times)
1258	813	0.06282	813	0.11885	+0.05603 (+1.90 times)
1160	726	0.11415	726	0.10249	−0.01166 (−0.10 times)
1090	685	0.06981	685	0.11732	+0.04751 (+1.68)
1034	518	0.08382	518	0.06483	−0.01899 (−0.29 times)
	460	1.26002	460	0.29245	−0.96757 (−4.30 times)

## Data Availability

Data are contained within the article and supplementary materials.

## Supplementary Materials

The following supporting information can be downloaded at https://www.mdpi.com/article/10.3390/toxins16070310/s1. Table S1: Applied kinetic models [37]; Table S2: Parameters for calculation of chronic daily intake set by USEPA [40]; Table S3: Reference dose (RfD) for different metals according to USEPA IRIS [39].
