# Supplementary material for: Kinetics and Mechanism of Cyanobacteria Cell Removal Using Biowaste-Derived Activated Carbons with Assessment of Potential Human Health Impacts
Source: Toxins (Basel). 2024 Jul 9;16(7):310. doi: 10.3390/toxins16070310 (PMC11281101; doi:10.3390/toxins16070310)
Supplement: Supplementary file 1 [file toxins-16-00310-s001.zip › toxins-3039744-supplementary.pdf]

Supplementary materials

Table S1. Applied kinetic models [62].

Model	Equation	Parameter
Pseudo-first order model	$q_t = q_e(1 - e^{-k_1 t})$	q_e (cells/mg): adsorption capacity at equilibrium<br>q_t (cells/mg) adsorption capacity at time t<br>k_1 (min^{-1}): the pseudo-first order rate constant
Pseudo-second order model	$q_t = \frac{t}{\left(\frac{1}{k_2 q_e^2}\right) + (\frac{t}{q_e})}$	q_e (cells/mg): adsorption capacity at equilibrium<br>q_t (cells/mg) adsorption capacity at time t<br>k_2 (mg/cells/min): the pseudo-second order rate constant
Elovich model	$q_t = \frac{1}{b}\ln(ab) + \frac{1}{b} lnt$	a (cells/g/min): initial adsorption rate<br>b (g/cells): extent of surface coverage

Table S2. Parameters for calculation of chronic daily intake set by USEPA [65].

Exposure parameters	Unit	Value	Reference
Metal concentration (C_w)	mg/L	/	/
**Ingestion**			
Ingestion rate of water (*IR)*	L/day	2.5	[66]
Exposure frequency (*EF)*	day/year	350	[66]
Exposure duration (*ED)*	year	70	[67,68]
Body weight (*BW)*	kg	70	[69]
Averaging time (*AT*)	day	25,550	[67]
**Dermal**			
Exposed skin surface area (*SA*)	cm^2	19,652	[63,70]
Specific dermal permeability coefficient (*Kp*)	cm/h	Chemical specific	[66]
Volumetric conversation factor (*CF*)	cm^3/L	10^{-3}	/
Exposure time (*ET*)	h/day	0.67077	[66]

Table S3. Reference dose (RfD) for different metals by USEPA IRIS [64].

Heavy metals	***RfD* [mg/kg/day]**
Cd	5.0×10^{-4}
Fe	7.0×10^{-1}
Ni	2.0×10^{-2}
Pb	3.5×10^{-3}
Hg	1.6×10^{-4}
Zn	3.0×10^{-1}
Cu	4.0×10^{-2}
As	3.0×10^{-4}
Cr	3.0×10^{-3}
Mn	2.4×10^{-2}
